# CT based PSI blocks for osteotomies around the knee provide accurate results when intraoperative imaging is used

**DOI:** 10.1186/s40634-021-00357-8

**Published:** 2021-06-26

**Authors:** Peter Savov, Mara Hold, Maximilian Petri, Hauke Horstmann, Christian von Falck, Max Ettinger

**Affiliations:** 1 Department of Orthopedic Surgery, Hannover Medical School, Anna-von-Borries-Strasse 1-7, 30625 Hanover, Germany; 2 Institute for Radiology, Hannover Medical School, Carl-Neuberg-Straße 1, 30625 Hanover, Germany

**Keywords:** PSI, High tibial osteotomy, Distal femoral osteotomy, Correction osteotomy

## Abstract

**Purpose:**

Correction osteotomies around the knee are common methods for the treatment of varus or valgus malalignment of the lower extremity. In recent years, patient specific instrumentation (PSI) guides were introduced in order to enhance the accuracy of these procedures. The purpose of this study was to determine the accuracy of CT based PSI guides for correction osteotomies around the knee of low volume osteotomy surgeons and to evaluate if CT based PSI blocks deliver a high degree of accuracy without using intraoperative fluoroscopy.

**Methods:**

Two study arms with CT based PSI cutting blocks for osteotomies around the knee were conducted. Part one: A retrospective analysis of 19 osteotomies was made in order to evaluate the accuracy in the hands of a low volume surgeon on long-leg radiographs. Part two: A cadaveric study with 8 knees was performed for the purpose of analyzing the accuracy without using intraoperative fluoroscopy on pre- and postoperative CT scans. Hip-Knee-Ankle angle (HKA), lateral distal femoral angle (LDFA) and medial proximal tibial angle (MPTA) were analyzed. The mean absolute delta (∂) between the planned and postoperative parameters were calculated. The accuracy of both study arms were compared.

**Results:**

Part one: The mean MPTA ∂, LDFA ∂ and HKA ∂ was 0.9°, 1.9° and 1.5°, respectively. Part two: The mean MPTA ∂ and LDFA ∂ was 3.5° and 2.2°, respectively. The mean ∂ of MPTA is significantly different between clinical patients with fluoroscopic control and cadaveric specimens without fluoroscopic control (*P* < 0.001). All surgeries were performed without complications such as a hinge fracture.

**Conclusion:**

The clinical use of PSI guides for osteotomies around the knee in the hands of low volume surgeons is a safe procedure. The PSI guides deliver a reliable accuracy under fluoroscopic control whereas their non-use of intraoperative fluoroscopy leads to a lack of accuracy. The use of fluoroscopic control during PSI guided correction osteotomies is highly recommended.

**Level of evidence:**

IV – Retrospective and experimental Study

## Introduction

Correction osteotomies around the knee are common methods for the treatment of varus or valgus malalignment of the lower extremity. The choice of procedure depends on the location of the deformity [1, 17]. The intention is to reduce the one sided overload of the femoral-tibial joint [8]. Over or under correction can lead to persistent pain and a paradoxical joint line as well as biomechanical issues [23]. Various techniques have been described in the literature to achieve the desired correction [12, 19, 28]. Classic manual techniques use a standardized methodology with conventional instruments in order to determine the extent of the axis change [19]. However, those techniques reach their limit especially in beginners or low volume surgeons [18, 30, 32].

In the past decade, computer navigation was introduced to enhance the accuracy of correction osteotomies. An advantage is the real-time control of the alignment as well as the possibility to evaluate the correction more precisely in the sagittal plane. Certain publications focusing on this topic present more precise results for navigation assisted correction osteotomies compared to traditional manual methods [3, 12, 24, 26]. However, navigation assisted correction osteotomies do not show consistently high precision throughout the literature. Furthermore, the extended operating times as well as additional costs are reported drawbacks [30].

An alternative to the surgical procedures mentioned above are patient-specific instruments (PSI). These custom-made cut blocks allow three-dimensional correction of both the tibia and the femur. The majority of the cutting blocks are made using computer tomography (CT)-based 3D models of the patient’s individual anatomy. The CT includes images of the femoral head, the knee and the ankle in order to calculate not only the hip-knee-ankle angle (HKA) but also the medial proximal tibial angle (MPTA), the lateral distal femoral angle (LDFA) and posterior tibial slope (PTS). Based on these parameters, the plate position, the resection plane, the degree of correction and the screw positions are virtually planned preoperatively. Initial proof of concept studies showed an accuracy of less than 1° or 2° on average (range: ± 1°) [9, 21, 31]. In contrast to the use of PSI for total knee arthroplasty [20], their use for correction deformities is based on multiple fluoroscopy shots during the operation in order to secure their correct position.

This study aims to answer the following questions:

1. Part 1: Are CT based PSI guides for correction osteotomies around the knee in the hands of low volume osteotomy surgeons a safe procedure?
2. Part 2: Can CT based PSI blocks deliver a high degree of accuracy without using intraoperative fluoroscopy?

The primary hypothesis is that CT-based PSI guides for correction osteotomies around the knee of low volume osteotomy surgeons provide a high accuracy. The secondary hypothesis is that the high accuracy can be conducted without intraoperative fluoroscopic control.

## Material and methods

### Part one

#### Material

Between January 2018 and August 2019 there were 30 osteotomies around the knee performed in 29 patients using PSI guides with the Activmotion plate (Newclip Technics, Haute-Goulaine, France) by one low volume osteotomy surgeon. The surgeon had performed less than 10 procedures per year before 2018. Inclusion criteria for this radiological analysis were the presence of standard antero-posterior (AP), lateral and full weight bearing long leg radiographs. Only patients with OW-HTO, CW-DFO or OW-DFO were included. Patients with derotating osteotomies were excluded.

#### Surgical technique

**Fig. 1:**
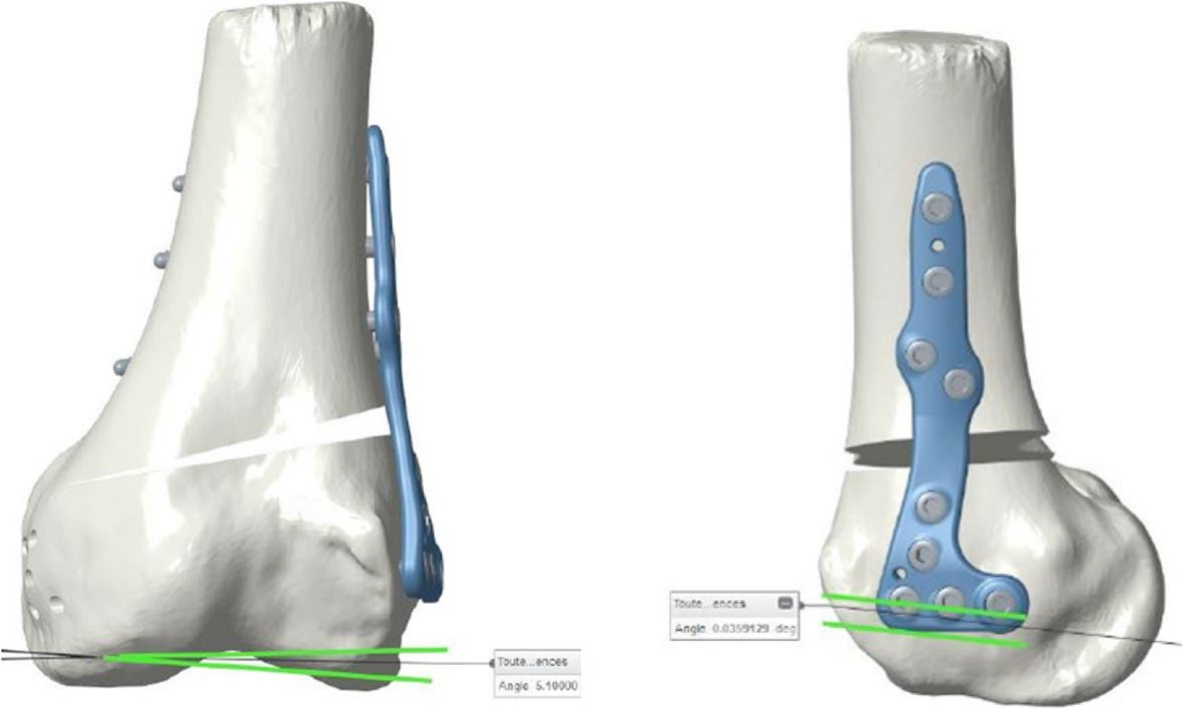
Within the 3D planning process, the plate position, the resection plane, the degree of correction, as well as the screw positions were virtually planned

**Fig. 2:**
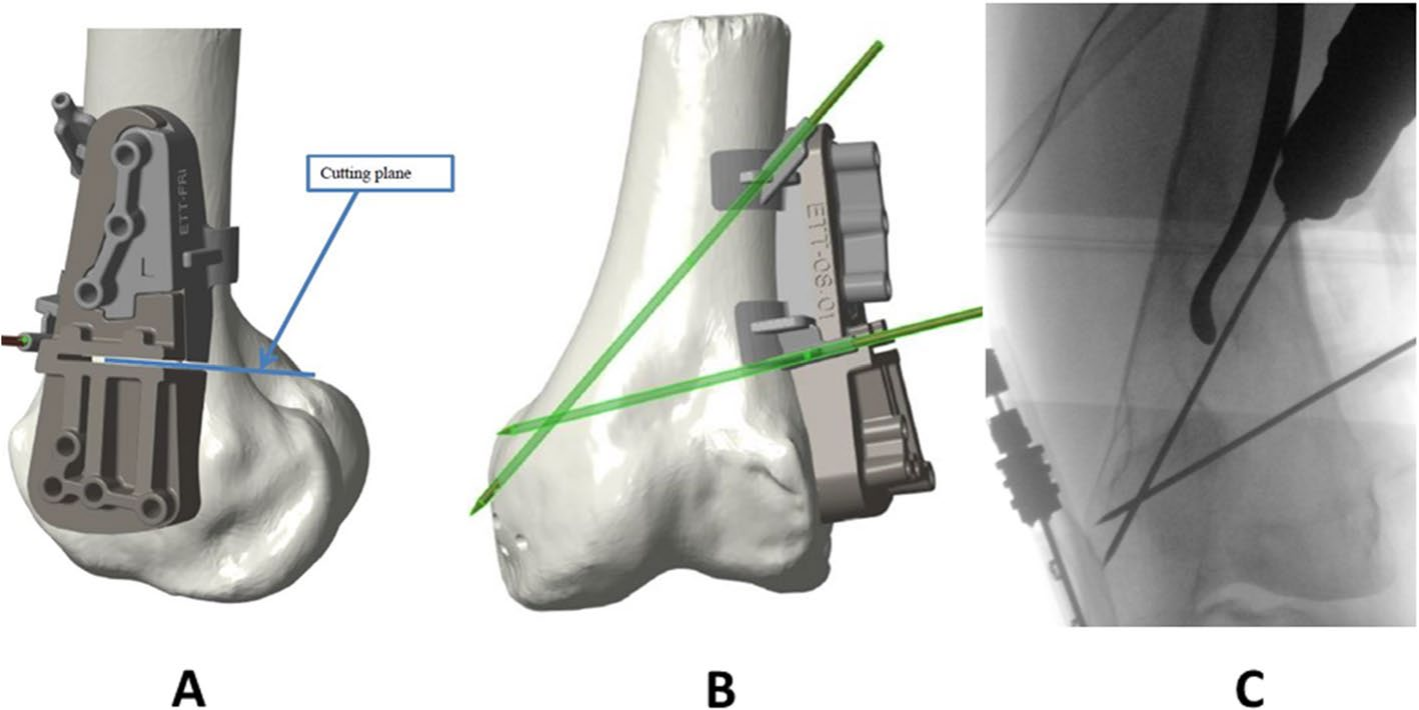
Intraoperatively, the PSI guide was fixed with two K-wires (**A** + **B**) under fluoroscopy control (**C**) in order to control the correct positioning of the PSI guide

**Fig. 3:**
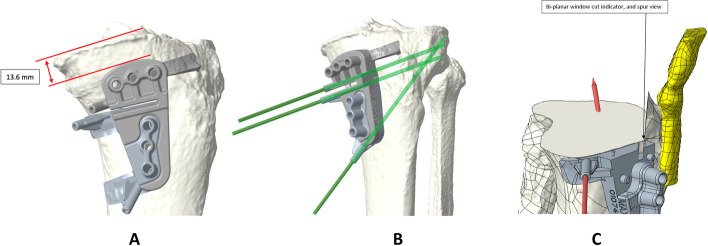
Intraoperatively, the PSI guide was fixed with three K-wires (**A** + **B**) in order to control the correct positioning of the PSI guide. After performing the osteotomy, the cutting guide is divided and the biplanar cut can be performed (**C**)

OW-HTO, CW-DFO and OW-DFO models were used to virtually position the manufacturers specific plates. Within the 3D planning process, the plate position, the resection plane, the degree of correction as well as the screw positions were virtually planned (Fig. 1). Intraoperatively, the PSI guide was fixed with two or three K-wires under fluoroscopy control in order to control the correct positioning of the PSI guide (Figs. 2 and 3). The oblique k-wire served as a hinge protection. The medial collateral ligament and the pes anserinus are protected with retractors and were not harmed. After confirmation of the planned position, the holes for the plate were pre-drilled. With this step, the plate position was pre-set prior to the osteotomy. Changing the opening or closing angle was therefore no longer possible. In the next step, the osteotomy was performed using the guided slot of the PSI leaving the oblique K-wire in place in order to secure the hinge. Subsequently, the cutting block is divided and the guided biplanar cut is performed (Fig. 3C). The osteotomy was then successively opened respectively closed until the screw holes of the plate fitted with the pre-drilled holes of the bone. In the final step, the plate was fixed. Only the position of the PSI cutting guide and screw length were controlled with fluoroscopy.

### Part 2

#### Material

In this cadaver study, both medial OW-HTO and lateral OW-DFO were performed on eight long leg cadaveric specimens (four right, four left/ Science Care, Phoenix, Arizona, USA). The BMI of the 6 male and 2 female specimens was lower than 28 kg/m^2^. The specimens had an age between 58 and 72 years. A CT according to the company’s protocol was performed. Therefore, images of the femoral head, the knee and the ankle were included. For acquisition hip, knee and ankle were scanned with 2.5 mm and for the reconstruction the knee was scanned with 0.625 mm slice thickness. For the OW-HTO an arbitrary 5° valgus correction was planned. For OW-DFO an arbitrary 5° varus correction was planned. The same plate as in part one was used for both the HTO and the DFO.

#### Surgical technique

**Fig. 4:**
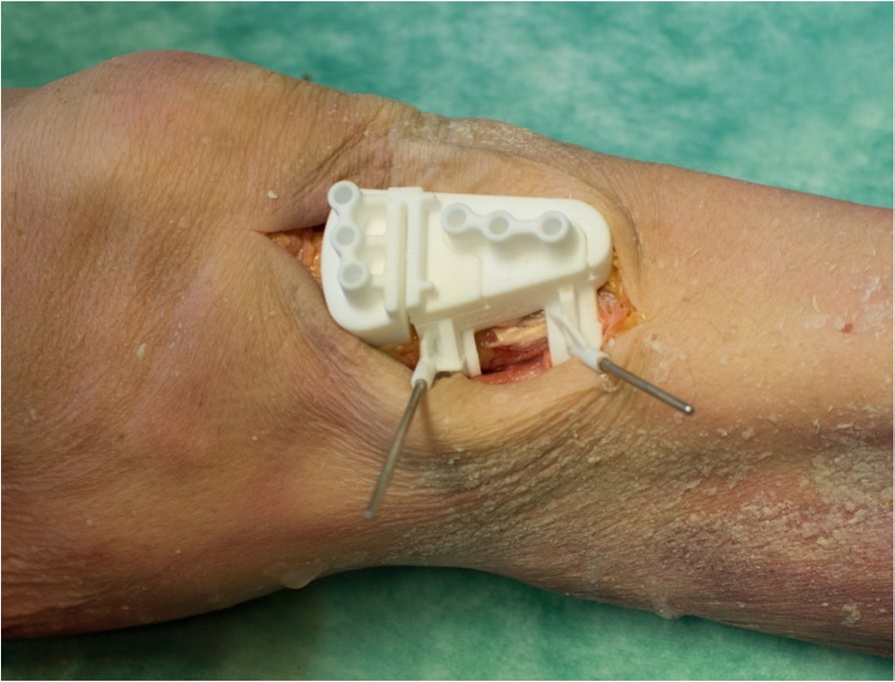
3D printed cutting guide fixed with two K-wires. The soft tissue was removed from the bone at the attachment points. No further tissue was removed or released in order to simulate an in-vivo situation to the highest degree

Soft tissue was removed from the bone at the attachment points for the PSI guide. There was no further tissue removed or released in order to mimic an in-vivo situation to the highest degree (Fig. 4). The 3D printed cutting blocks were adapted to the individual anatomy of the specimen without fluoroscopy and only with respect to the individual plan. For orientation the distance to the joint gap was measured and compared to the preoperative plan (Figs. 2 and 3). The cutting guide was placed without fluoroscopic control. The rest of the surgery was performed as described in the clinical part (part one) of the study.

### Parameters

**Fig. 5:**
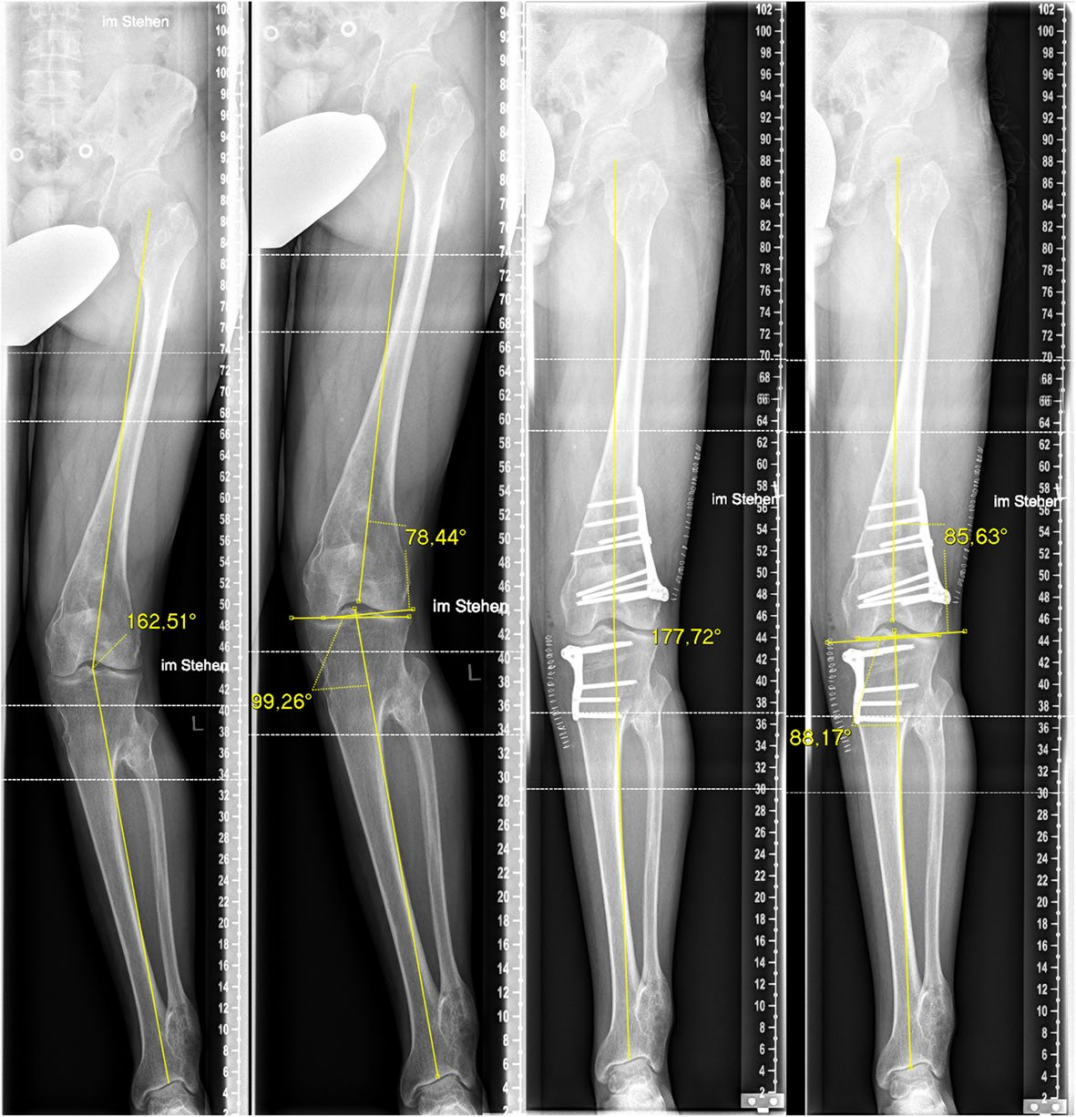
The accuracy of the procedure was defined as the delta (∂) between planned correction and postoperative alignment measured on full weight bearing long leg radiographs (∂ HKA **A** + **C**, ∂ MPTA **B** + **D**, ∂ LDFA **B** + **D**)

All included patients from study arm part one received standard antero-posterior (AP), lateral and long leg radiographs postoperatively 5 days after surgery and full weight bearing long leg radiographs after 3 months. The accuracy of the procedure was defined as the delta (∂) between planned correction and postoperative alignment (∂ HKA, ∂ MPTA, ∂ LDFA / Fig. 5) measured on full weight bearing long leg radiographs at 3 months follow-up. After the surgery, the total radiation exposure due to intraoperative fluoroscopy was recorded in cGy*cm2. For study arm part two the accuracy of the procedure was defined as the ∂ between planned correction and postoperative CT scans (∂ MPTA, ∂ LDFA). All measurements were performed by one orthopedic senior consultant, one orthopedic resident and one experienced radiologist.

### Statistical analysis

The statistical analysis was performed with GraphPad Prism 7 (GraphPad Inc., San Diego, CA, USA). To calculate differences between the mean values, the student´s t-test was used. To calculate differences between the medians, the Wilcoxon-u-test was used. The level of significance was set to 0.05. To determine the inter observer reliability (IOR), the interclass correlation coefficient (ICC) was calculated. A post hoc analysis was made computing achieved power. The effect side was calculated with the means and sample size of ∂ MPTA and ∂ LDFA. Alpha was set to 0.05. The post hoc power for ∂ MPTA and ∂ LDFA was 0.99 and 0.08, respectively.

## Results

Part 1: After in- and exclusion criteria: Three patients were excluded due to missing long leg x-rays postoperatively, while two patients with derotating osteotomies were excluded as well. In total, 19 PSI based osteotomies were included into this retrospective radiological analysis (Table 1). 11 extremities were treated with an OW-HTO, while 4 extremities were treated with a CW-DFO and 2 extremities received an OW-DFO. 2 extremities received a double-level osteotomy (OW-HTO + OW-DFO). The mean age was 42.6 years (SD ± 13.7), there were 13 male and 6 female patients. The mean BMI was 27.5 kg/m^2^ (SD ± 4.1).

**Table 1:** Fluoroscopic data of all patients with the preoperative and postoperative alignment as well as the planned parameter and the absolute deviation between the postoperative radiological outcome and the planned values

**ID**			**Preoperative**			**Planned**			**Postoperative**			**Absolut delta**			**Fluoroscopy**	
	**HTO/DFO**	**Procedure**	**MPTA**	**LDFA**	**HKA**	**MPTA**	**LDFA**	**OLA**	**MPTA**	**LDFA**	**HKA**	**MPTA**	**LDFA**	**HKA**	cGy*cm^**2**^	**Pictures**
**1**	HTO	OW	80.65	85.38	171	90		185	90.67	85.38	185	0.67		0	31.69	21
**2**	HTO	OW	85.29	89.69	173.31	90		180	89.3	89.69	179.97	0.7		0.03	52.8	12
**3**	HTO	OW	84.19	90.11	172.51	90		180	90.22	90.11	179.67	022		0.33	54.3	6
**4**	HTO	OW	84.44	92.81	174.11	90		180	90.01	92.81	179.28	0.01		0.72	26.9	18
**5**	HTO	OW	84.73	89.34	172.73	90		180	88.99	89.34	178.7	1.01		1.3	7.7	13
**6**	HTO	OW	85.16	93.35	170.28	91		180	93.28	93.35	179.83	2.28		0.17	24.91	19
**7**	HTO	OW	84.83	86.28	172.45	90		180	90.7	86.28	179.15	0.7		0.85	33	21
**8**	HTO	OW	85.66	91.35	168.43	90		179	89.1	91.35	177.25	0.9		1.75	12.93	9
**9**	HTO	OW	82.98	91.58	172.99	90		180	88.56	91.58	177.11	1.44		2.89	14.68	12
**10**	HTO	OW	80.32	89.7	169.6	88		180	88.64	89.7	179.25	0.64		0.75	13.41	16
**11**	HTO	OW	83.84	89	175.56	88		179	87.72	89	177.24	0.28		1.76	4,69	9
**12**	HTO + DFO	OW + CW	99.59	79.13	197.99	87	89	180	87.98	85.61	182.48	0.98	3.39	2.48	43	52
**13**	HTO + DFO	OW + CW	95.14	81.99	193.24	87	87	180	88.47	88.78	180.76	1.47	1.78	0.76	23.62	49
**14**	DFO	CW	89.42	80.31	189.16		88	180	89.42	87.98	177.05		0.02	2.95	53.19	
**15**	DFO	CW	92.79	85.4	182.29		90	180	92.79	86.33	183.14		3.67	3.14	45.1	3
**16**	DFO	CW	90.11	82.43	187.29		90	180	90.11	87	182.92		3	2.92	117.1	2
**17**	DFO	CW	91.9	81.05	191.95		88	180	91.9	88.82	179.34		0.82	0.66	12.86	8
**18**	DFO	OW	89.96	83.13	172.24		90	180	89.96	90.9	176.66		0.9	3.34	33.8	37
**19**	DFO	OW	89.75	84.66	174.98		90	180	89.75	92.29	179.22		2.29	0.78	26.7	32
	**Mean**		**87.41**	**86.67**	**178.01**	**89.31**	**89.00**	**180.2**	**89.87**	**89.28**	**179.7**	**0.86**	**1.98**	**1.45**	**33.28**	**18.83**
	**SD**		**4.89**	**4.35**	**8.96**	**1.3**	**1.19**	**1.21**	**1.51**	**2.42**	**2.31**	**0.60**	**1.33**	**1.16**	**24.89**	**14.28**
	**Min**		80.32	79.13	168.43	87	87	179	87.72	85.38	176.7	0.01	0.02	0	4.69	2
	**Max**		99.59	93.35	197.99	91	90	185	93.28	93.35	185	2.28	3.67	3.34	117.1	52

HKA below 180° is defined as varus alignment

*HKA* hip-knee-ankle angle, *MPTA* media proximal tibial angle, *LDFA* lateral distal femoral angle, *HTO* high-tibial osteotomy, *DFO* distal femoral osteotomy, *OW* open wedge, *CW* closed wedge, *SD* standard deviation

The mean planned MPTA and LDFA were 89.31° (SD ± 1.3°) and 89° (SD ± 1.19°), respectively. The mean postoperative MPTA and LDFA was 89.87° (SD ± 1.51°) and 89.28° (SD ± 2.42°), respectively. The mean ∂ between planned HKA and postoperative HKA was 1.45° ± 1.16°, the mean absolute ∂ between planned MPTA and postoperative MTPA was 0.86° ± 0.6° and the mean absolute ∂ between planned LDFA and postoperative LDFA was 1.98° ± 1.33°. The mean radiation exposure per procedure was 33.28° ± 24.89 cGy*cm2. All findings are summarized in Table 1. The ICC for part 1 is 0.962 (95% CI 0.915 – 0.994). All surgeries were performed without complications such as hinge fractures.

Part 2: All surgeries were performed without complications such as a hinge fracture. The mean ∂ between planned MPTA and postoperative MTPA was 3.47° ± 1.07° and the mean ∂ between planned LDFA and postoperative LDFA was 2.18° ± 1.9°. All findings are summarized in Table 2. The ICC for part 2 is 0.912 (95% CI 0.865 – 0.975).

**Table 2:** Fluoroscopic data of all specimens with the preoperative and postoperative alignment as well as the absolute deviation between the postoperative radiological outcome and the planned correction of 5° for the HTO and DFO

Specimen	Preoperative		Postoperative		Absolute delta	
	**LDFA**	**MPTA**	**LDFA**	**MPTA**	**LDFA**	**MPTA**
L172563_left	84.07	87.19	92.95	90.04	3.88	2.15
L172563_right	83.23	87.46	93.04	87.4	4.81	5.06
L172616_left	86.38	86.6	89.84	87.9	1.54	3.7
L172616_right	87.03	86.78	96.55	88.58	4.52	3.2
L172619_left	82.56	95.34	88.36	98.2	0.8	2.14
L172619_right	80.59	90.31	86.19	NA	0.6	NA
L172621_left	88.06	90.57	93.18	91.33	0.12	4.24
L172621_right	86.98	88.45	90.8	89.67	1.18	3.78
**Mean**	**84.86**	**89.09**	**91.36**	**90.45**	**2.18**	**3.47**
**SD**	**2.63**	**2.95**	**3.25**	**3.67**	**1.90**	**1.07**
**Min**	80.59	86.60	86.19	87.40	0.12	2.14
**Max**	88.06	95.34	96.55	98.20	4.81	5.06

*MPTA* media proximal tibial angle, *LDFA* lateral distal femoral angle

The mean ∂ MPTA was significantly different between clinical patients and cadaveric specimens (*P* < 0.001, 95% CI: -3.4 to -1.9 / Table 3). The mean ∂ LDFA was not significantly different between clinical patients and cadaveric specimens (*p* = 0,813, 95% CI: -2 to 1.6 / Table 3).

**Table 3:** Statistical analysis of the absolute delta between the mean and median of the planned alignment and the radiological outcome

	**Patients**	**Specimens**	***p*—Value**	**95% CI**
Mean ∂ MPTA in °	0.86 (± 0.60)	3.47 (± 0.99)	< 0.001*	-3.4 to -0.19
Median ∂ MPTA in °	0.7	3.7	< 0.001*	
Mean ∂ LDFA in °	1.98 (± 1.33)	2.18 (± 1.78)	n.s	-2 to 1.6
Median ∂ LDFA in °	2	1.4	n.s	

^*^Statistical significant

## Discussion

The most important finding of this study is the fact that the clinical use of PSI guides for correction osteotomies around the knee delivers a reliable accuracy under fluoroscopic control in the hands of low volume osteotomy surgeons. Thus, there is no accuracy for the learning curve. In contrast to that, omitting fluoroscopic control consequences in a lack of accuracy.

The clinical part of our study showed a high degree of accuracy in OW-HTO in the hand of a low volume osteotomy surgeon. Our results with a mean ∂ MPTA of 0.86° are comparable to the findings reported by Chaouche et al. who evaluated a high degree of accuracy for PSI guides in OW-HTO. They reported a mean ∂ HKA and ∂ MPTA of 1° ± 0.95° and 0.54° ± 0.63° in a cohort of a hundred patients. Furthermore, no complications specific to PSI were observed [5]. These values are comparable to recently published studies that showed no learning curve in terms of accuracy for PSI based osteotomies around the knee [15, 21].

However, publications on DFO osteotomies using PSI cutting blocks are rare as well [14, 27]. Two studies reported data concerning the accuracy of PSI guides in opening wedge distal femur varization. Jacquet et al. reported a significantly improved accuracy concerning the postoperative alignment compared to a conventional group (0.43° ± 0.50° vs 3.95° ± 1.64°) [14]. Elattar et al. [10] and Victor et al. [31] presented similar findings for OW-DFO and considered PSI guides as reliable tools for this procedure whereas Shi et al. reported a high degree of accuracy for CW-DFO in 33 knees as well.

One potential benefit of a PSI block for osteotomies around the knee is a guided placement of a K-wire in order to protect the lateral hinge during the procedure (Figs. 2 and 3). A hinge fracture incidence of about 30% is reported throughout the literature [7, 29]. A stable hinge is considered as a crucial part in osteotomies around the knee since the hinge is important for preservation of the correction as well as the bony consolidation of the osteotomy [16]. Practically, by intersecting the K-wire into the cutting plane at the planned hinge location, the cutting depth would be limited [6]. Further, the K-wire helps to preserve the lateral hinge during the opening of the osteotomy. Dessyn et al. [6] evaluated this effect in a biomechanical approach testing fresh frozen cadavers with and without a PSI guided K-wire for lateral hinge protection. Their biomechanical study revealed that the maximum load to breakage and the maximum permissible displacement were, respectively, 880% and 260% higher during the opening of the OWHTO by using K-wires compared to the non-K-wire control group. This confirms the mechanical advantage of using a K-wire for both stabilization and protecting the hinge during OW-HTO. Equal clinical findings were recently published by Gulagaci et al. who demonstrated that during OW-HTO a K-wire in the lateral hinge location reduced the occurrence of intraoperative lateral hinge fractures [11]. These findings are comparable to our results since there were no hinge fractures seen in both parts of our study.

Besides the clinical accuracy, the second research question was to evaluate the use of PSI cutting guides without intraoperative fluoroscopy like PSI blocks for TKA. We evaluated a mean dose area product (DAP) of about 33 cGy*cm2 and about 18 fluoroscopic images per patient within our clinical evaluation. The mean DAP of a standard thorax p.a. x-ray is 9 cGy*cm^2^ and of a lower spine x-ray is 140 cGy*cm^2^ [25]. To evaluate the actual radiation effect for the patient the effective dose has to be considered. However, this can be neglected for the knee joint due to the very low conversion factor to the effective dose (0,1 µSv/cGy*cm^2^) [13]. More important is the radiation dose reduction for the surgical team. Furthermore, no other study focusing on PSI guided osteotomies reported the intraoperative fluoroscopy radiation dose before. Thus, a comparison to current literature is not possible. However, Jacquet et al. reported a mean of 4.3 fluoroscopy images after a 9 cases learning curve for a PSI guided technique [15]. Further, Gulagaci et al. [11] reported a mean of 5.1 fluoroscopic images per procedure in a series of 60 knees using the same PSI guides. Pérez-Mañanes et al. report a mean of 8 fluoroscopic images for HTO and Arnal-Burró et al. a mean of 6 fluoroscopic images for DFO per procedure with do-it-yourself cutting guides [2, 22]. One explanation for these results might be differences concerning the level of experience of the surgeon by using these specific PSI blocks.

The results of our cadaveric approach reveal that omitting intraoperative fluoroscopy leads to a lack of accuracy. The results of our cadaveric analysis are significantly different compared to Donnez et al. [9] whose cadaveric study, with the exact same approach as ours, revealed an accuracy of a ∂ MPTA of 0.2° compared to a mean ∂ MPTA of 3.47° in our cadaveric study. This significant difference might be explainable due to the fact that Donnez et al. removed all soft tissues except for the patellae tendon insertion. In contrast to that, we only removed soft tissue from the bone at the attachment points for the PSI guide in order to mimic the in vivo situation as best as possible (Fig. 5). While referencing only with respect to the bony landmarks without fluoroscopy the soft tissue irritation is in our point of view the main issue and leads to the minor accuracy of the cutting guides. Further, the conflict of approach size and minimal invasive surgery cannot be controlled without objective information like x-ray data.

Several study limitations have to be mentioned. Part one of this study is a retrospective analysis without any patient outcome measurements in a small series of patients. Further, no control group was analyzed. The retrospective approach might introduce a potential selection bias. We used postoperative long leg radiographs in order to calculate the preoperative to postoperative ∂ of the HKA, MPTA and LDFA. However, Boonen et al. reported a good validity comparing measurements of long leg radiographs to 3D CT-scans [4]. In part two a small number of specimens were used in order to evaluate the effect of no fluoroscopic imaging. Moreover, not all specimens had a sufficient deformity to be considered as candidates for an osteotomy around the knee. In order to reduce a planning bias, OW-HTOs were planned with an arbitrary of 5° valgus correction and OW-DFOs with an arbitrary of 5° varus correction. However, lateral hinge fractures may not have occurred due to the correction of only 5°. Further, the amount of the correction has not yet been studied for the accuracy of PSI blocks. Nevertheless, the ∂ of accuracy compared to current literature and our clinical cohort is significantly different.

## Conclusion

The clinical use of PSI guides for correction osteotomies around the knee under fluoroscopic control is a safe procedure in the hand of low volume osteotomy surgeons. In contrast to that, omitting fluoroscopic control consequences in a lack of accuracy. The use of fluoroscopic control during PSI guided correction osteotomies around the knee is highly recommended.
